# Risk factors to guide Terson syndrome screening after aneurysmal subarachnoid Hemorrhage

**DOI:** 10.1007/s10143-025-03956-6

**Published:** 2026-01-16

**Authors:** Ilies Djebbara, Sune Munthe, Troels Halfeld Nielsen, Lejla Islamagič

**Affiliations:** 1 Department of Neurosurgery, Odense University Hospital, Odense, Denmark; 2 Faculty of Health Sciences, University of Southern Denmark, Odense, Denmark; 3 Department of Clinical Research, University of Southern Denmark, Odense, Denmark; 4 BRIDGE (Brain Research – Interdisciplinary Guided Excellence), University of Southern Denmark, Odense, Denmark

**Keywords:** Terson syndrome, Aneurysmal subarachnoid hemorrhage, Predictive model, Risk stratification, Ophthalmologic complications

## Abstract

Terson syndrome is defined as intraocular hemorrhage secondary to aneurysmal subarachnoid hemorrhage (aSAH). Despite clinical relevance, it remains under-recognized and is associated with poorer neurological and visual outcomes. Early detection is difficult in critically ill patients unable to report symptoms. We aimed to identify independent predictors and develop a clinically usable risk score for targeted ophthalmologic screening. We conducted a retrospective cohort study of 220 adult aSAH patients admitted to Odense University Hospital (Sept 1, 2018-Jan 21, 2025), of whom 89 underwent ophthalmologic screening. Terson syndrome was defined as any intraocular hemorrhage on examination. Predictors were selected using least absolute shrinkage and selection operator (LASSO) logistic regression, with multivariable analysis and internal validation via bootstrapping. A point-based risk score was derived and compared to a literature-based model using discrimination, calibration, information criteria, and decision curve analysis. Sensitivity analyses included Firth logistic regression and inverse probability weighting. Among 220 patients, 89 (40.5%) were screened; 31 had Terson syndrome (14.1% overall; 34.8% of screened). Three predictors were identified: male sex (OR 10.9), higher Hunt-Hess grade (OR 3.0 per grade), and anterior communicating artery aneurysm (OR 8.6; all *p* < 0.05). The model demonstrated strong discrimination (AUC 0.862; 95% CI, 0.787–0.937), good calibration, and outperformed the literature-based comparator (AUC 0.769). A 0–7 point-based score stratified risk from < 25% to > 80%, with superior net benefit. Sensitivity analyses confirmed robustness. This three-variable model offers a pragmatic tool for early Terson syndrome detection and may reduce preventable visual morbidity through targeted screening.

**Supplementary Information:**

The online version contains supplementary material available at 10.1007/s10143-025-03956-6.

## Introduction

Terson syndrome (TS) was first described by Moritz Litten in 1881 and later characterized by Albert Terson [1, 2]. TS is defined as intraocular hemorrhage occurring in association with acute intracranial bleeding, most commonly aneurysmal subarachnoid hemorrhage (aSAH) [3]. Although classically involving hemorrhage into the vitreous body, TS may affect the subhyaloid, subretinal, and retinal layers as well [3–5]. These ocular hemorrhages are thought to arise from sudden intracranial pressure (ICP) elevations, leading to retinal venous hypertension and vessel rupture [3]. In patients with aSAH, TS is relatively common, but often under-recognized. Reported incidence rates vary widely (from approximately 5%−42%) due to inconsistent screening practices and clinical awareness [3, 6–8]. Many critically ill aSAH patients cannot report visual symptoms, and ophthalmologic screening is not routinely performed in acute care [9, 10]. This diagnostic awareness is clinically important because the presence of TS is associated with increased morbidity and may indicate more severe underlying brain injury [4, 11, 12].

Previous studies have identified several factors associated with increased risk of TS in aSAH, including higher Hunt-Hess grade (HHG), higher World Federation of Neurosurgical Societies (WFNS) grade, lower Glasgow Coma Scale (GCS) score, larger aneurysm size (e.g., ≥ 5 mm), and treatment modality (endovascular coiling versus surgical clipping) [6, 13, 14]. However, these studies were typically small or single-center, lacked systematic TS screening, and did not undergo external validation [15]. Moreover, a recent systematic review by Al-Shalchy & Al-Wassiti [16] underscores the need for validated, clinically usable tools. To the best of our knowledge, no studies have directly compared traditional predictor-based models with data-driven selection methods in well-characterized cohorts.

Timely recognition of TS is clinically relevant because persistent vitreous hemorrhage can result in irreversible visual impairment, whereas early vitrectomy has been associated with substantially better visual outcomes [17]. Improved risk stratification may therefore translate directly into preserved long-term vision and functional recovery.

To address these limitations and support more systematic early identification, we retrospectively analyzed a screened cohort of aSAH patients to identify independent predictors of TS and develop a concise, risk-guided model aimed at improving clinical triage and targeted ophthalmologic screening.

## Methods

### Study design

We conducted a retrospective cohort quality improvement study at Odense University Hospital (OUH), Denmark. We queried the hospital electronic patient record systems for all patients admitted with aSAH between September 1, 2018, and January 21, 2025. Patients were eligible if they were ≥ 18 years, had confirmed aSAH, and underwent an ophthalmologic examination during hospitalization. We excluded patients with SAH secondary to arteriovenous malformations or genetic conditions, traumatic SAH, inter-hospital transfers, or death prior to eye examination. After applying these criteria, 89 aSAH patients with documented ophthalmologic screening remained (out of 220 initially reviewed). Screening was not systematic but rather guided by clinical suspicion, documented vision complaints, or clinical severity, reflecting real-world triage practices. To improve transparency and assess potential selection bias, we included all 220 patients in the descriptive analysis of baseline characteristics. Table 1 presents demographic and clinical variables for the entire aSAH cohort, including those not screened.

### Data collection and definitions

Two investigators independently extracted data on predefined variables. The primary outcome was TS, defined as any intraocular hemorrhage (vitreous, subhyaloid, intraretinal, or subretinal) documented on ophthalmologic exam. Absence of such a finding was recorded as no TS. We recorded demographic, clinical, and treatment-related variables, including age, sex, admission neurological status, vascular risk factors, and aneurysm characteristics (rupture location and aneurysm size in mm). Detailed variable definitions are provided in Table 1.

**Table 1:** Baseline characteristics of patients with aneurysmal subarachnoid hemorrhage

	**Total number of patients(n=220)**		**Patients assessed by ophthalmologists(n=89)**				**P-value**
	**n**	**(%)**	**No Terson(n=58)**	**(%)**	**Terson(n=31)**	**(%)**	
**General characteristics**							
*Age (median, 95% CI)*							
Male	59 (55-63)		55.00 [51.00;61.00]		55.50 [51.50;65.00]		0.620
Female	60 (59-64)		57.50 [52.00;61.50]		60.00 [52.00;65.00]		0.707
*Sex*							
Male	62	28.2	10	45.45	12	54.55	**0.048**
Female	158	71.8	48	71.64	19	28.36	**0.048**
**Demographics**							
Hypertension	79	35.9	21	60.00	14	40.00	0.551
Hypercholesterolemia	24	10.9	5	55.56	4	44.44	0.714
Diabetes Mellitus	8	3.6	2	40.00	3	60.00	0.337
Migraine	14	6.4	4	80.00	1	20.00	0.654
Connective-tissue disorders	4	1.8	1	33.33	2	66.67	0.277
**Smoking status**							
Current smoker	68	30.9	14	66.67	7	33.33	1.000
Previous smoker	40	18.2	10	62.50	6	37.50	1.000
Never smoked	51	23.2	18	78.26	5	21.74	0.202
Unknown	61	27.7	16	55.17	13	44.83	0.255
**Alcohol consumption**							
Alcohol (weekly consumption > 10 units)	60	27.3	15	60.00	10	40.00	0.695
No alcohol	44	20.0	11	68.75	5	31.25	0.966
Unknown	76	34.5	20	55.56	16	44.44	0.180
**Mortality**							
Died within 1-year	19	8.6	6	75.00	2	25.00	0.708
Died within 2-years	2	0.9	1	100.00	0	0.00	1.000
**Initial GCS**							
15	75	34.1	24	85.71	4	14.29	**0.008**
13-14	63	28.6	21	75.00	7	25.00	0.280
9-12	13	5.9	2	22.22	7	77.78	**0.008**
6-8	19	8.6	6	85.71	1	14.29	0.414
3-5	50	22.7	5	29.41	12	70.59	**0.002**
**Variables related to the aneurysm or complications to aSAH**							
EVD	114	51.8	31	63.27	18	36.73	0.847
1 aneurysm	180	81.8	53	67.95	25	32.05	0.259
2 or more aneurysms	40	18.2	5	45.45	6	54.55	0.259
*Size < 1 cm (largest diameter)	169	76.8	45	66.18	23	33.82	0.923
Size ≥ 1 cm (largest diameter)	42	19.1	8	53.33	7	46.67	0.448
ACOM	90	40.9	16	45.71	19	54.29	**0.004**
PCOM	25	11.4	12	80.00	3	20.00	0.242
ACA	2	0.9	0	0.00	0	0.00	1.000
MCA	42	19.1	9	60.00	6	40.00	0.870
ICA	33	15.0	11	78.57	3	21.43	0.363
AChorA	0	0.0	0	0.00	0	0.00	1.000
PICA	11	5.0	6	85.71	1	14.29	0.414
PCLA	9	4.1	3	100.00	0	0.00	0.549
SCA	3	1.4	2	100.00	0	0.00	0.541
VA	3	1.4	1	100.00	0	0.00	1.000
BA	18	8.2	5	62.50	3	37.50	1.000
Intracranial haematoma in relation to aneurysm	68	30.9	12	46.15	14	53.85	**0.030**
Re-bleeding during hospitalization	39	17.7	7	70.00	3	30.00	1.000
Complication to endovascular procedure	17	7.7	2	50.00	2	50.00	0.608
Seizures at debut	35	15.9	5	38.46	8	61.54	0.061
Seizures during hospitalisation	10	4.5	1	100.00	0	0.00	1.000
Coiling	158	71.8	43	64.18	24	35.82	0.933
Clipping	50	22.7	13	72.22	5	27.78	0.670
Other	12	5.5	2	50.00	2	50.00	0.608
Clinical vasospasms	58	26.4	12	54.55	10	45.45	0.343
Radiological vasospasms	58	26.4	10	50.00	10	50.00	0.177
Transcranial Doppler vasospasms	26	11.8	2	25.00	6	75.00	**0.020**
**Length of ICU-stay**							
<10 days	90	40.9	28	80.00	7	20.00	**0.033**
>10 days	130	59.1	30	55.56	24	44.44	**0.033**
**Grading systems**							
*mFG*							
1	16	7.3	5	83.33	1	16.67	0.661
2	13	5.9	6	85.71	1	14.29	0.414
3	83	37.7	27	75.00	9	25.00	0.168
4	108	49.1	20	50.00	20	50.00	**0.013**
*WFNS*							
1	76	34.5	24	85.71	4	14.29	**0.008**
2	52	23.6	15	75.00	5	25.00	0.434
3	10	4.5	6	75.00	2	25.00	0.708
4	26	11.8	7	43.75	9	56.25	0.090
5	56	25.5	6	35.29	11	64.71	**0.010**
*HHG*							
1	35	15.9	12	92.31	1	7.69	**0.029**
2	81	36.8	26	81.25	6	18.75	**0.031**
3	26	11.8	7	53.85	6	46.15	0.540
4	23	10.5	6	46.15	7	53.85	0.214
5	55	25.0	7	38.89	11	61.11	**0.019**

Data are presented as n (%) for categorical variables and median (95% CI) for continuous variables. P-values were calculated using Fisher's exact test or Chi-square test for categorical variables and Mann-Whitney U test for continuous variables.

*P*-values compare Terson vs No Terson among ophthalmologically assessed patients

Significant *p*-values (<0.05) are shown in bold

*aSAH*, aneurysmal subarachnoid hemorrhage; *CI*, confidence interval; *EVD*, external ventricular drain; *GCS*, Glasgow Coma Scale; *HHG*, Hunt and Hess Grade; *ICU*, intensive care unit; *mFG*, modified Fisher Grade; *WFNS*, World Federation of Neurological Surgeons scale; *ACOM*, anterior communicating artery; *PCOM*, posterior communicating artery; *ACA*, anterior cerebral artery; *MCA*, middle cerebral artery; *ICA*, internal carotid artery; *AChorA*, anterior choroidal artery; *PICA*, posterior inferior cerebellar artery; *PCLA*, posterolateral artery; *SCA*, superior cerebellar artery; *VA*, vertebral artery; *BA*, basilar artery

### Statistical analyses

We followed STROBE guidelines for observational studies. All candidate predictors were first analyzed univariately: categorical variables by Chi-square or Fisher’s exact test, and continuous variables by Student’s t-test (if approximately normally distributed) or Mann-Whitney U test (if not). Variables with *p* < 0.05, or known clinical relevance, were considered for multivariable models. To build a model, we applied LASSO-penalized logistic regression with 10-fold cross-validation to optimize the regularization parameter (λ). The optimal λ resulted in a subset of predictors with nonzero coefficients. These variables were entered into a standard multivariable logistic regression (binomial distribution, logit link) to estimate adjusted odds ratios (ORs) and 95% confidence intervals (CIs) for TS. Model performance was assessed using the area under the receiver operating characteristic curve (AUC), Akaike and Bayesian Information Criteria (AIC/BIC), and likelihood ratio tests. Calibration was evaluated using the Hosmer-Lemeshow goodness-of-fit test (lfit, group(10) in Stata), with *p* > 0.05 indicating acceptable fit. Collinearity among predictors was assessed using variance inflation factors (VIFs). Internal validation was performed using bootstrap resampling with 1,000 iterations to assess predictor stability. Based on the final LASSO-selected multivariable model, we derived a point-based clinical risk score by converting β-coefficients into integer-weighted values. Given the number of events per variable (~ 10 EPV rule), the use of a three-variable final model was statistically appropriate. In parallel, we constructed a comparator “literature-based” logistic regression model. This model included four established TS risk factors: WFNS grade, early seizure occurrence, aneurysm size ≥ 5 mm, and treatment with endovascular coiling [3, 13, 14, 18]. We fitted this model on our cohort data. Finally, we compared the two models’ performance by their AUC, AIC, BIC, and overall explanatory power. Decision curve analysis (DCA) was performed to assess the clinical utility of the final risk model. Predicted probabilities from the logistic regression were evaluated across threshold probabilities ranging from 10% to 80%. Net benefit was calculated by weighing true positives against false positives to simulate the impact of using the model to guide ophthalmologic screening decisions. All analyses were performed in Stata/MP 18.0, and two-tailed *p* < 0.05 was considered significant.

### Sensitivity analyses

To assess the robustness of our model to potential bias, we conducted two sensitivity analyses. First, we applied Firth’s penalized logistic regression to reduce small-sample bias and address potential separation. Second, to account for the non-random screening process (only 89/220 patients underwent ophthalmologic evaluation), we applied inverse probability weighting (IPW) using stabilized weights. A logistic regression model predicting the probability of being screened was constructed using the same predictors included in the final model: Sex, HHG, and ACom aneurysm location. These weights were used in a weighted logistic regression model restricted to the screened subgroup.

## Results

### Main findings

**Fig. 1:**
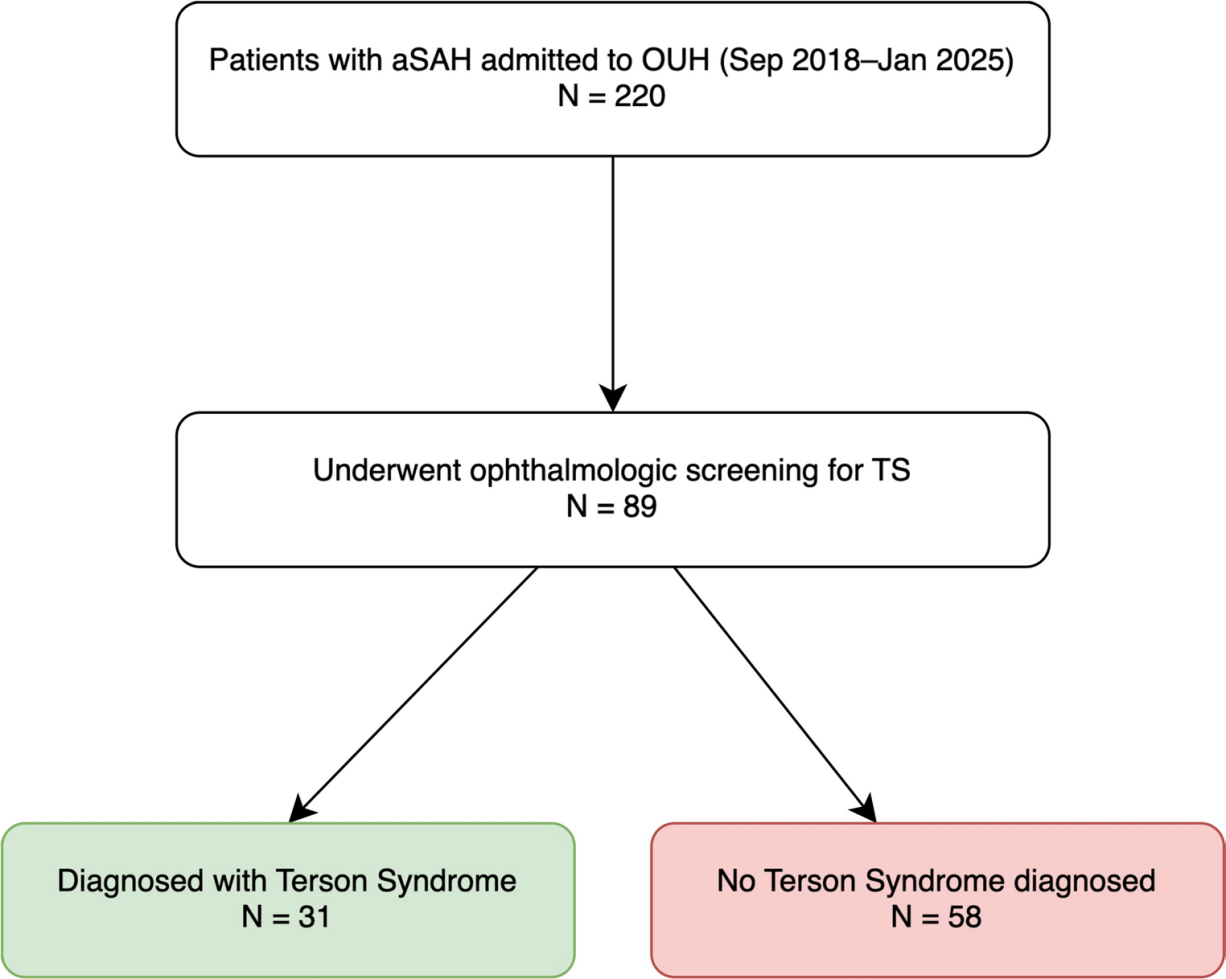
Diagram depicting screening, exclusion, and inclusion of patients with aneurysmal subarachnoid hemorrhage (aSAH) at Odense University Hospital. Of 220 patients, 89 met inclusion criteria and underwent ophthalmologic screening for TS

Among the 220 patients with aSAH during the study period, 89 (40%) underwent an ophthalmologic examination. Compared to unscreened patients, those who underwent ophthalmologic evaluation were more likely to present with higher HHG, lower GCS, anterior circulation aneurysms, and male sex (Table 1). This suggests a non-random screening pattern influenced both by clinical presentation and by pragmatic factors such as personnel availability and whether ophthalmologic examinations were requested, introducing potential selection bias that reflects real-world clinical practice. Recognizing this bias is important for interpreting model generalizability. TS was diagnosed in 31 of 89 screened patients (34.8%), corresponding to 14.1% of the total cohort (31/220). The study inclusion process is summarized in Fig. 1, outlining patient eligibility, exclusions, and the final analysis cohort.

In univariate analyses, several variables were significantly associated with TS status (Table 1). Male patients were more likely to develop TS (54.5% of examined males vs. 28.4% of females, p = 0.025). Higher admission HHG was strongly associated with TS: 61% of patients with grade 5 had TS compared to only 8% with grade 1 (p = 0.009 for grade 5 vs. grade 1). Patients with ruptured anterior communicating artery (ACom) aneurysms also had a higher incidence of TS (54%) than those with other aneurysm locations (29%, p = 0.002). By contrast, age and common vascular risk factors (hypertension, diabetes, smoking) did not differ significantly between groups.

The LASSO procedure, using 10-fold cross-validation and the 1-standard error rule, identified three key predictors: sex, HHG, and ACom aneurysm. These were entered into a logistic regression model, which fit the data well and was concise (Table 2). Each predictor remained independently significant (all p < 0.05): male sex (OR 10.9), HHG (OR 3.0 per grade), and ruptured ACom aneurysm (OR 8.6 vs. other sites). No other clinical or radiographic variables added significantly once these three factors were included. Sensitivity analyses adding variables such as modified Fisher grade or treatment modality showed no meaningful improvement in AIC or model discrimination, consistent with the LASSO selection.

**Table 2:** Final multivariable logistic regression model identifying independent predictors of Terson syndrome after aneurysmal subarachnoid hemorrhage (aSAH)

Predictor	$\:\beta\:$- coefficient	Odds Ratio (OR)	95% CI for OR	*P*-value
Male sex	2.39	10.95	[2.95–47.67]	0.001
HHG	1.11	3.03	[1.60–5.73]	< 0.001
ACom aneurysm	2.15	8.63	[2.43–30.70]	0.001

Data shown include regression coefficients (β), adjusted odds ratios (OR), and corresponding 95% confidence intervals (CI) for each predictor. Predictors were selected using LASSO regression and confirmed via internal bootstrap validation

All listed predictors are statistically significant (*P* < 0.05)

*HHG* Hunt–Hess grade, *ACom* anterior communicating artery, *CI* confidence interval, *OR* odds ratio

**Fig. 2:**
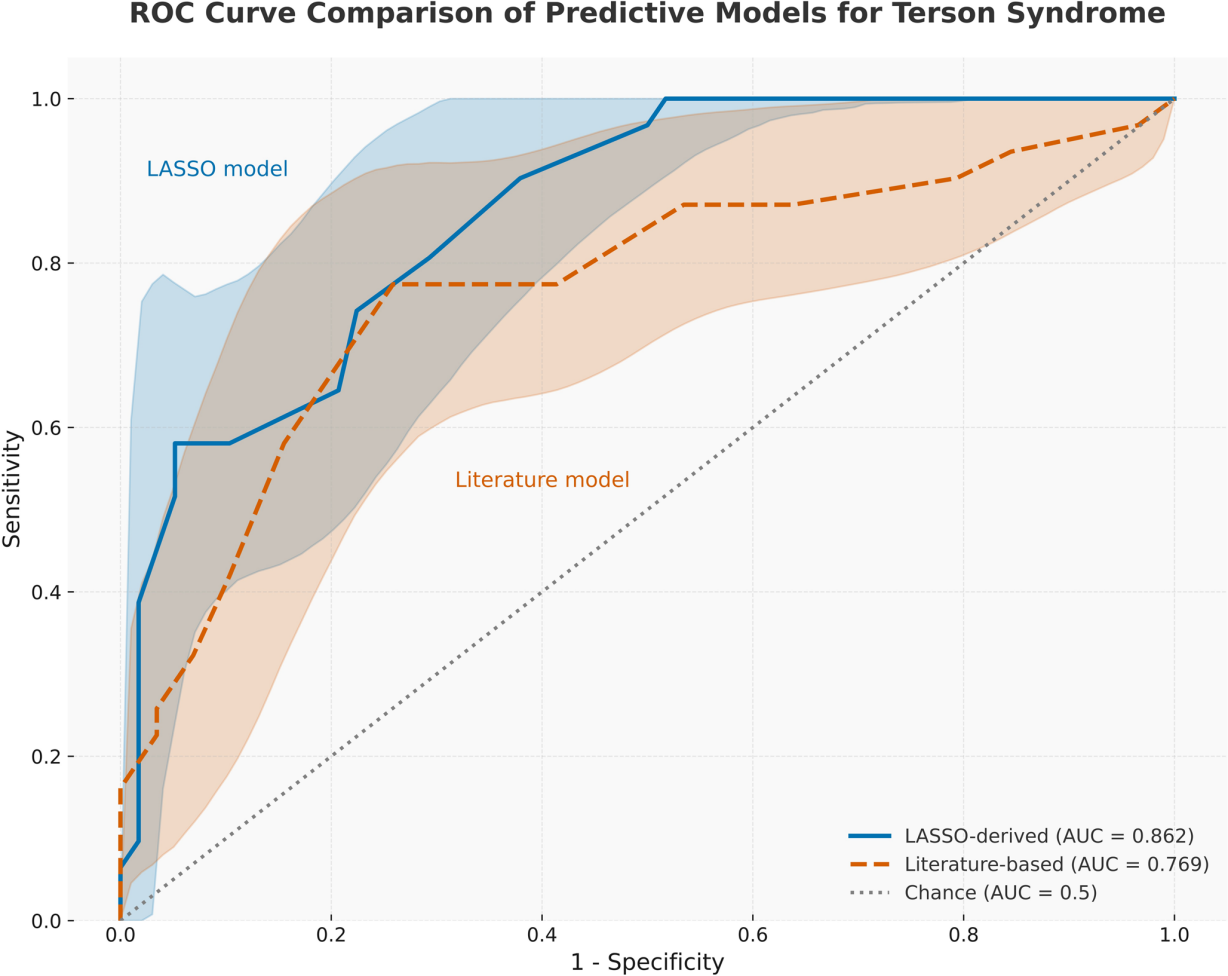
Receiver operating characteristic (ROC) curves comparing the performance of the LASSO-derived predictive model (blue curve) and the literature-based model (orange dashed curve) for identifying Terson syndrome (TS) after aneurysmal subarachnoid hemorrhage (aSAH). The area under the curve (AUC) was 0.862 for the LASSO-derived model compared to 0.769 for the literature-based model, demonstrating superior discriminative capability. The diagonal dotted gray line represents chance prediction (AUC = 0.5). A higher AUC indicates better model accuracy

**Fig. 3:**
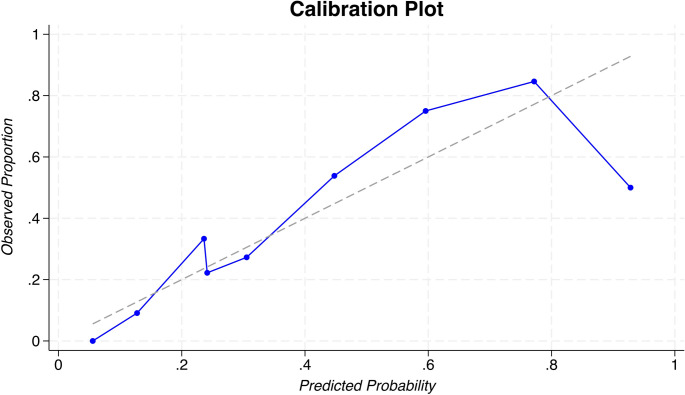
Calibration Plot Calibration plot of the LASSO-derived model for predicting Terson syndrome (TS). Predicted probabilities were plotted against observed frequencies. The dashed line represents perfect calibration. The close overlap between observed and predicted risks confirms good model calibration, with slope ≈1.00 and intercept ≈0.00

We compared this data-driven model to the literature-based model of WFNS grade, seizures, aneurysm size ≥ 5 mm, and endovascular coiling. The literature-based model achieved AUC 0.769 (95% CI: 0.657–0.881) with AIC = 104.17 and BIC = 116.61. In contrast, our three-variable model achieved AUC 0.862 (95% CI: 0.787–0.937) with lower AIC (82.9) and BIC (92.8), indicating better fit despite fewer variables (Table 3; Fig. 2). These results indicate that our model achieved superior discrimination and goodness-of-fit compared to established models. Calibration was also acceptable: the Hosmer-Lemeshow test showed no evidence of lack of fit (χ² [7] = 7.60, p = 0.370), suggesting good agreement between predicted and observed risk. The calibration plot (Fig. 3) further demonstrated excellent alignment, with a slope of 1.00 and an intercept near 0.00, suggesting no systematic miscalibration.

**Table 3:** Comparative performance of the LASSO-derived predictive model versus a literature-based predictive model for Terson syndrome in patients with aneurysmal subarachnoid hemorrhage (aSAH)

Model	Included predictors	AIC	BIC	AUC (95% CI)
LASSO-derived model	Male sex, HHG, ACom aneurysm	82.86	92.81	0.862 (0.79–0.94)
Literature-based model	WFNS grade, seizure, aneurysm size ≥5 mm, endovascular coiling	104.17	116.61	0.769 (0.66–0.88)

Performance metrics provided include Akaike Information Criterion (AIC), Bayesian Information Criterion (BIC), and area under the receiver operating characteristic curve (AUC), alongside corresponding 95% confidence intervals (CI)

Lower AIC and BIC values indicate better model fit, whereas higher AUC values reflect improved model discrimination. The LASSO-derived model demonstrated superior overall performance compared to the literature-based model

*HHG* Hunt–Hess grade, ACom anterior communicating artery, *WFNS* World Federation of Neurosurgical Societies grade, *AIC* Akaike Information Criterion, *BIC* Bayesian Information Criterion, *AUC* Area Under the receiver operating characteristic Curve, *CI* confidence interval

Both Firth and IPW analyses confirmed the robustness of our primary findings. In the Firth logistic model, all three predictors—male sex (OR 4.03, *p* = 0.020), HHG (OR 2.18 per grade, *p* < 0.001), and ACom aneurysm (OR 4.15, *p* = 0.007)—remained statistically significant. Similarly, in the IPW-weighted regression, all predictors remained significant: male sex (OR 9.7, *p* = 0.004), HHG (OR 3.0 per grade, *p* < 0.001), and ACom aneurysm (OR 7.3, *p* = 0.001). These findings suggest the model’s associations are robust to both small-sample bias and potential selection bias due to non-random screening.

**Fig. 4:**
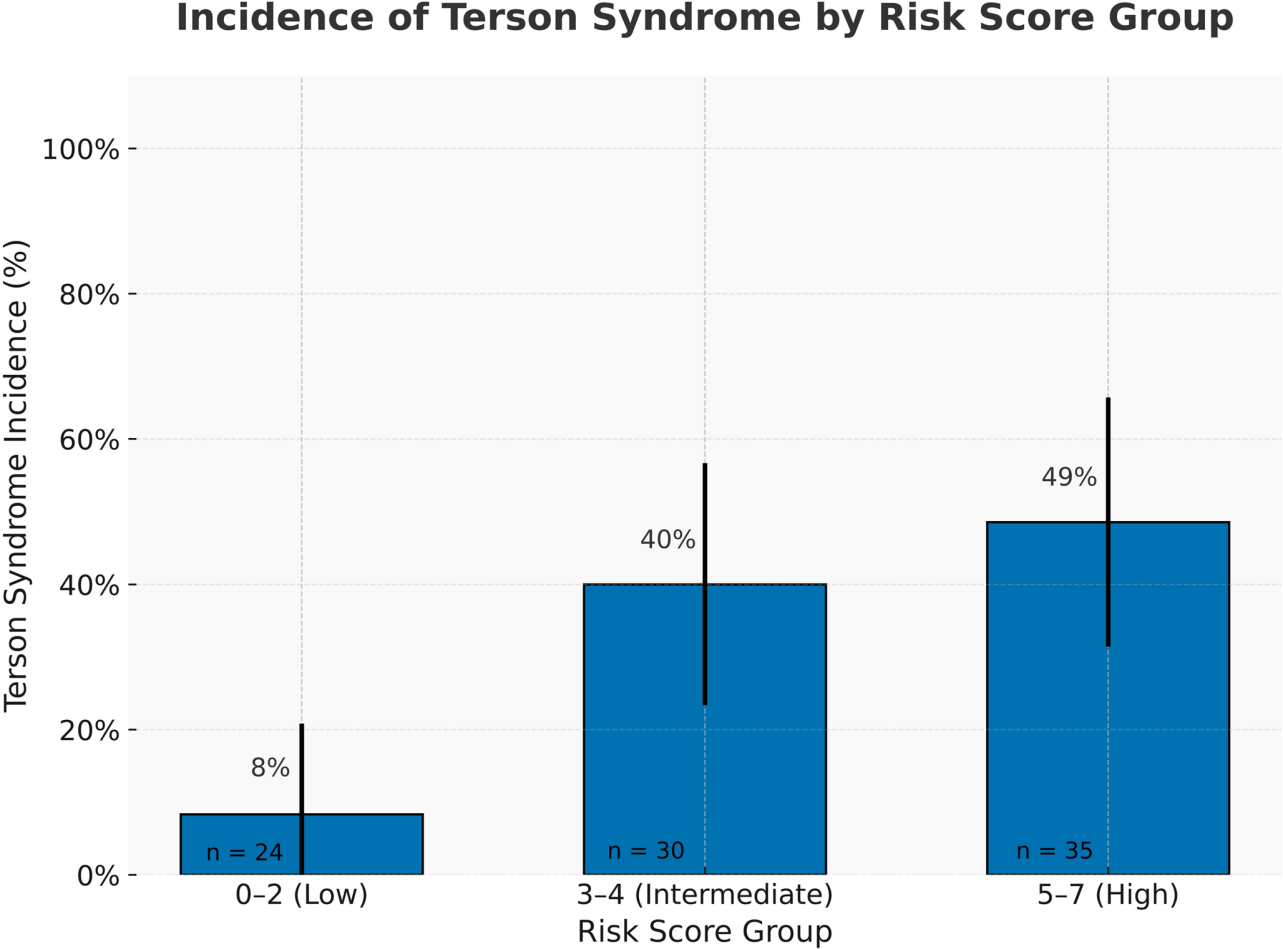
Incidence of Terson syndrome stratified by clinical risk score categories: low (0–2 points), intermediate (3–4 points), and high (5–7 points). The incidence of TS increases stepwise with increasing risk category. Bars represent the proportion of patients diagnosed with TS within each group, accompanied by 95% binomial confidence intervals. The sample size (n) for each group is indicated within each bar, emphasizing clinical applicability and sample representation

**Fig. 5:**
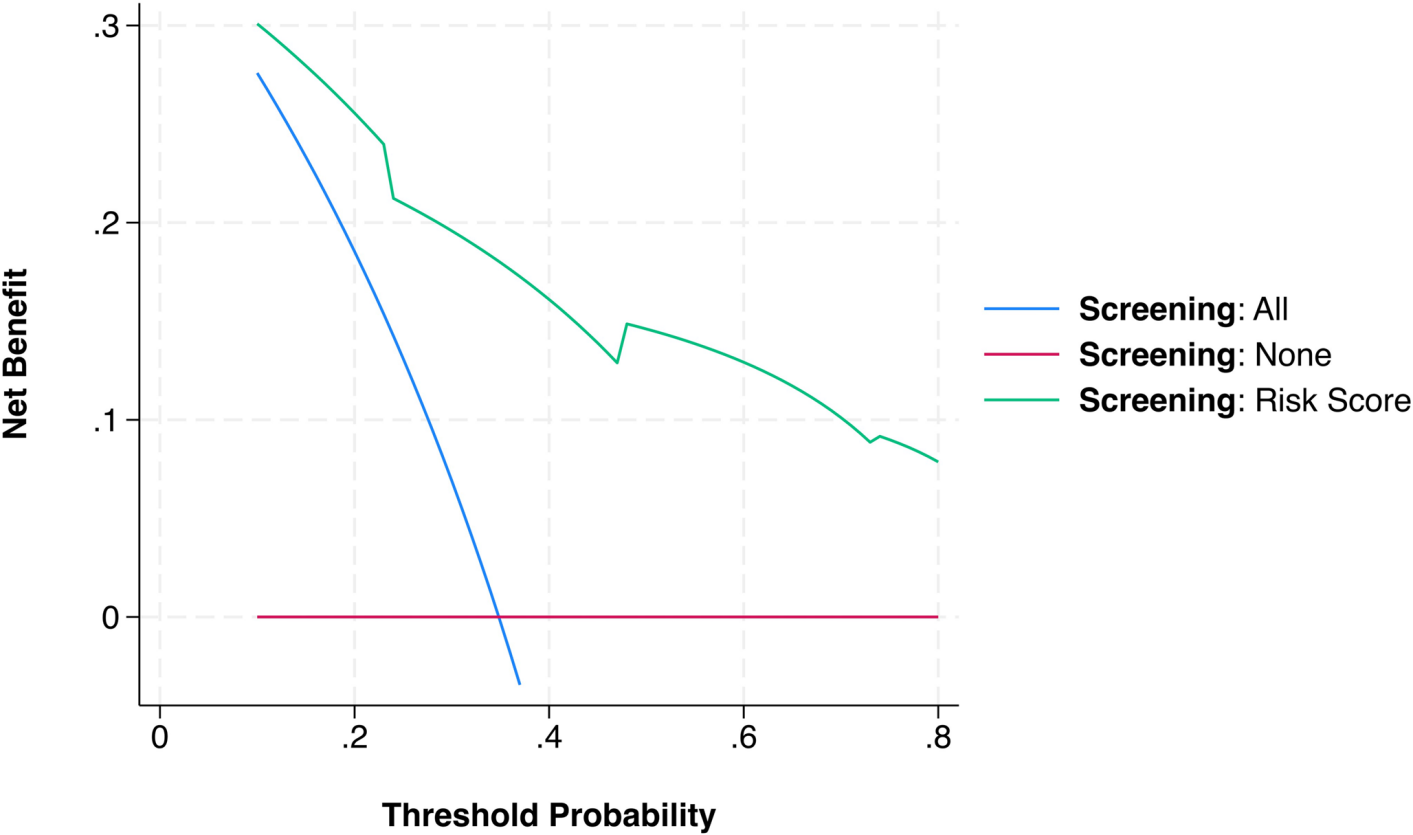
Decision curve analysis illustrating the clinical utility of the Terson syndrome risk score. The green curve demonstrates the net clinical benefit of employing the predictive model to guide targeted ophthalmologic screening across various threshold probabilities (ranging from 10% to 80%). The risk model provides greater net clinical benefit compared to universal screening ("Screen All," blue line) or no screening ("Screen None," red line) strategies. Net benefit quantifies the trade-off between correctly identifying TS cases and avoiding unnecessary screenings, reinforcing the pragmatic clinical advantage of the proposed risk stratification

Using the final model coefficients, we derived a 3-variable, point-based TS risk score based on model coefficients (Table 4). Points were assigned as follows: male sex = 2, each one-point HHG increment = 1, ACom aneurysm = 2 (score range 0–7). Higher scores predicted higher TS incidence. In our cohort, patients with scores 0–2 had TS rates 0–25%, scores 3–4 had rates 40–70%, and scores ≥ 5 had rates > 80% (100% at the top score). This stepwise increase (Fig. 4) supports the score’s discriminative capacity. To enhance clinical utility, we translated risk scores into estimated probabilities of TS occurrence based on observed incidence across score values. Table 5 provides a practical summary of predicted TS risk per total point score, with suggested clinical actions. This tool supports bedside decision-making and facilitates timely ophthalmologic referral. Furthermore, decision curve analysis (Fig. 5) demonstrated that the TS risk model provided greater net clinical benefit than both “screen all” and “screen none” strategies across a range of threshold probabilities (10% to 75%). This suggests that using the model to guide ophthalmologic screening could reduce unnecessary exams while maintaining detection of high-risk TS cases. Net benefit was maximized at intermediate risk thresholds (~ 30–60%), consistent with a selective screening approach.

**Table 4:** Point-based clinical risk score for predicting Terson syndrome (TS) in patients with aneurysmal subarachnoid hemorrhage (aSAH)

Predictor	β-Coefficient	OR	Assigned Points
Male	2.39	10.95	+ 2
HHG	1.10	3.00	1 + per grade
ACom	2.15	8.60	+ 2

This scoring system was derived from the final multivariable logistic regression model using LASSO-selected predictors and validated through internal bootstrap resampling. Scores range from 0 to 7, with higher scores indicating a greater risk of developing TS

Clinically, patients scoring ≥3 points should be considered at significantly elevated risk and prioritized for ophthalmologic screening within the first 72 hours of admission. External validation of this simplified clinical tool is recommended before routine implementation

*HHG* Hunt–Hess grade, *ACom* anterior communicating artery, *OR* odds ratio

**Table 5:** Estimated probability of Terson syndrome (TS) according to the total clinical risk score (range 0–7)

Total Risk Score	Estimated TS Probability	Suggested Clinical Action
0	~ 5%	Screening likely unnecessary
1	~ 10%	Consider deferred screening
2	~ 20%	Low risk - screen if additional concerns
3	~ 40%	Moderate risk - screening recommended
4	~ 65%	Elevated risk - prioritize for screening
5	~ 80%	High risk - strong indication for screening
6–7	> 85–90%	Very high risk - urgent screening indicated

Probabilities are based on observed incidence rates in the derivation cohort. Suggested clinical actions are provided to guide ophthalmologic referral and prioritization in patients admitted with aneurysmal subarachnoid hemorrhage (aSAH).

Patients scoring ≥3 points should undergo timely ophthalmologic evaluation, with higher scores indicating progressively urgent screening needs. External validation is recommended to confirm these thresholds before widespread clinical adoption

## Discussion

In this retrospectively screened aSAH cohort, we identified three independent risk factors for Terson syndrome: male sex, higher HHG grade on admission, and ruptured ACom aneurysm. Beyond supporting the hypothesis that TS is driven by surges in intracranial pressure, our findings offer a clinically useful approach for stratifying risk and prioritizing ophthalmologic assessment in the acute neurocritical care setting. Our findings also underscore that identifying TS is not only prognostically relevant but therapeutically actionable: early diagnosis can facilitate timely ophthalmologic monitoring and, when indicated, vitrectomy, which has been shown to significantly improve long-term visual acuity [17]. Patients with more severe hemorrhages (higher grades) or certain aneurysm locations (ACom) may experience greater pressure surges, predisposing them to intraocular bleeding [5, 19]. Our predictive model using these three factors achieved strong discrimination (AUC 0.862), outperforming a traditional model of previously reported risk factors. Importantly, the model also demonstrated good calibration in internal validation, supporting its clinical applicability. This concise, data-driven model enables early identification of high-risk patients and offers a practical framework for TS screening in critically ill individuals unable to report symptoms. In settings where routine fundoscopy is unfeasible, it may serve as a triage tool prompting timely ophthalmologic consultation or bedside ultrasound. This model may be especially valuable in sedated or intubated ICU patients, where symptom-driven screening is not feasible. Structured screening using the score may reduce the risk of missed diagnoses while avoiding the burden of universal screening.

Our results reinforce prior evidence that greater hemorrhage severity, reflected by high HHG or WFNS grades, is a strong predictor of TS, likely due to associated acute ICP surges and venous pressure transmission [4, 6, 7, 13, 14, 18]. Given the time-sensitive nature of TS-related vision loss, early identification in high-risk individuals, those with elevated HHG, male sex, and ruptured ACom aneurysms, could facilitate timely ophthalmologic evaluation, including non-invasive bedside tools such as ocular ultrasound, and prevent irreversible visual impairment [10, 14, 15, 20, 21].

In our study, early post-hemorrhage seizures were not an independent predictor of TS after controlling for hemorrhage severity, although seizures have been identified as a risk factor in some larger series [13]. We also found that aneurysm location plays a role: rupture of an ACom aneurysm was associated with significantly higher odds of TS, consistent with findings by Joswig et al. [5] and supported by a recent systematic review by Al-Shalchy and Al-Wassiti [16], which identified anterior circulation aneurysms, particularly ACom, as having a disproportionately higher association with TS. Furthermore, ACom aneurysms were strongly associated with TS, potentially due to their proximity to the optic apparatus and greater pressure transmission during rupture. This aligns with prior studies linking anterior circulation aneurysms to increased TS risk [5, 16, 22]. Notably, our analysis identified male sex as a significant independent risk factor for TS. This contrasts with several earlier series suggesting female predominance [7, 13]. However, population-based data from South Korea also reported a higher relative incidence of TS in men (1.10% vs. 0.71% in women) [15]. Biological explanations may include sex-related differences in vascular wall integrity, collagen/elastin composition, or hormonal influences on rupture dynamics [15]. It is also conceivable that differences in rupture biomechanics or treatment pathways contribute [15, 23]. We tested a reduced model excluding sex, which reduced discrimination and calibration, suggesting that sex meaningfully improved predictive performance in our cohort. Nevertheless, this variable should be interpreted cautiously and requires confirmation in multicenter validation [23]. Another possible explanation might be socio-behavioral factors, such as delayed health-seeking behaviors more frequently observed in men, leading to worsened clinical status upon presentation. This disparity may reflect population differences, residual confounding, or diagnostic bias. Given the lack of systematic screening, underdiagnosis in women cannot be excluded [15]. To definitively clarify these findings, future research should incorporate larger, multicenter studies to thoroughly explore and validate this sex-related discrepancy. Further investigation in larger patient samples will be necessary to confirm whether male sex truly confers an increased susceptibility to TS.

Our analysis also highlights the effectiveness of employing modern statistical methods like LASSO regression. Recent literature underscores that, particularly in moderate-sized clinical datasets, LASSO regression offers a robust approach to minimize overfitting while reliably identifying clinically relevant predictors [24, 25]. This methodological strength is crucial in conditions such as TS, where modest sample sizes are common due to under-recognition and limited screening.

Our inability to capture patient-reported visual symptoms - either due to critical illness or fragmented follow-up - reflects a broader challenge in aSAH care, where only 12–34% of TS cases are diagnosed without protocolized screening [14, 15]. This gap reinforces the need for objective tools like our risk score, which identifies high-risk patients warranting mandatory fundoscopy regardless of symptom reporting. In our cohort, 40% of TS cases lacked documented visual complaints at the time of diagnosis, consistent with prior reports of asymptomatic TS in up to 30% of SAH patients [13, 15]. Moreover, patients with high HHG or WFNS grades frequently require intubation and prolonged mechanical ventilation, precluding their ability to report visual disturbances. Furthermore, decision curve analysis demonstrated that the model offers greater net clinical benefit than indiscriminate or no screening across a range of thresholds, supporting its role as a pragmatic tool in routine care. In addition to internal validation via bootstrapping and decision curve analysis, we conducted Firth penalized logistic regression and inverse probability weighting (IPW) analyses to account for small-sample and selection bias. Both confirmed the stability and significance of the three predictors, further supporting the robustness and internal validity of our model. It is worth noting that odds ratios varied somewhat between statistical approaches, which is expected because LASSO penalization shrinks coefficients to reduce overfitting, whereas conventional logistic regression provides unpenalized estimates. Despite these numerical differences, the same predictors were consistently selected across models, underscoring their robustness.

### Clinical implications

Despite its prevalence and prognostic relevance, TS often remains undiagnosed, potentially due to the challenges of universal ophthalmologic screening in critically ill aSAH patients. This limitation further reinforces the need for a systematic, risk score-guided screening strategy that operates independently of subjective symptom reporting. In practice, we suggest that patients with a score ≥ 3 should undergo mandatory ophthalmologic screening, ideally within 72 h of admission. First-line assessment should be direct or indirect fundoscopy, with ocular point-of-care ultrasound (POCUS) as a validated bedside alternative in sedated or intubated patients [5, 9, 19, 26]. Optical coherence tomography may be considered where available. Embedding this threshold-driven approach into neurocritical care protocols could ensure systematic and symptom-independent detection of TS.

A threshold score ≥ 3 captures over 80% of TS cases while avoiding nearly 40% of unnecessary exams compared to universal screening. This targeted approach may reduce preventable visual morbidity and optimize resource allocation in neurocritical care.

### Limitations

The single-center, retrospective design and modest sample size (31 TS cases among 89 screened patients) limit the external validity and generalizability of our findings. While our study represents one of the largest screened cohorts to date for TS, external validation in multicenter, prospective datasets is essential to confirm generalizability and real-world performance of the score across diverse settings. Because ophthalmologic examinations were ordered pragmatically—depending not only on clinical suspicion but also on personnel availability and routines—this non-systematic screening may have affected representativeness, potentially inflating the observed TS prevalence and limiting generalizability. As such, while internally valid, our model requires prospective validation in multicenter cohorts with standardized screening. Existing TS studies are often small, single-center, or registry-based and rarely provide patient-level data (e.g., neurological grade, aneurysm location, ICP dynamics) [15]. These limitations preclude their use as formal external validation datasets, reinforcing the need for prospective, multicenter collaborations. The relatively small number of events also precluded a comprehensive exploration of potential interaction effects between predictors, and the possibility of unidentified effect modification cannot be excluded. Furthermore, the absence of continuous intracranial pressure monitoring and detailed neuroimaging restricts mechanistic insight into the pathophysiology of TS. Nonetheless, this study constitutes one of the largest single-center cohorts of ophthalmologically screened aSAH patients, and the use of penalized regression, bootstrapping, and decision curve analysis helped reduce overfitting and strengthen internal validity. Ultimately, prospective external validation in larger and more diverse populations is warranted to confirm the broader clinical utility of the model.

## Conclusion

In this cohort, male sex, higher HHG, and ruptured ACom aneurysm independently predicted Terson syndrome, forming the basis of a simple three-variable risk score with strong discriminative performance. While these findings require external validation, the model may enable targeted screening, improve early detection, and prevent missed diagnoses of a clinically significant complication.

### Future perspectives

Prospective multicenter studies are essential to externally validate our model predictors and assess their generalizability across diverse neurocritical care populations. Future research should also explore gender-specific biological, anatomical, and social factors underlying the observed association between male sex and increased TS risk. Standardized ophthalmologic screening within 72 h of aSAH onset, combined with strengthened interhospital data-sharing agreements, could minimize diagnostic variability and attrition bias. Furthermore, advanced neuroimaging techniques, such as MRI-based biomarkers, and machine learning approaches may offer deeper insights and optimize targeted TS screening strategies.

## Supplementary Information

Below is the link to the electronic supplementary material.

ESM 1 DOCX (33.6 KB)

## Data Availability

The datasets analysed during the current study are available from the corresponding author on reasonable request.

## Abbreviations

HHG: Hunt–Hess grade
ACom: anterior communicating artery
WFNS: World Federation of Neurosurgical Societies grade
AIC: Akaike Information Criterion
BIC: Bayesian Information Criterion
AUC: Area Under the receiver operating characteristic Curve
CI: confidence interval
